# Camel Milk Cannot Prevent the Development of Cow's Milk Allergy—A Study in Brown Norway Rats

**DOI:** 10.1002/mnfr.202200359

**Published:** 2022-12-05

**Authors:** Natalia Zofia Maryniak, Mette Halkjær Stage, Anne‐Sofie Ravn Ballegaard, Ana Isabel Sancho, Egon Bech Hansen, Katrine Lindholm Bøgh

**Affiliations:** ^1^ National Food Institute Technical University of Denmark Kgs. Lyngby DK‐2800 Denmark

**Keywords:** allergy prevention, animal model, camel milk, cow's milk allergy, food allergy, infant formula

## Abstract

**Scope:**

Currently there are no specific recommendations for the use of any particular infant formula in the prevention of cow's milk allergy (CMA). Recently, there has been an increasing interest in alternative infant formulas based on milk proteins from other sources than the cow, including milk from other mammalians such as goat, sheep, donkey, horse, and camel. Whereas these have been studied for their usability in CMA management, there are no studies of their CMA preventive capacity. Thus, the aim of this study is to evaluate whether camel milk can prevent CMA and vice versa.

**Methods and results:**

The capacity of camel milk in preventing CMA and vice versa is evaluated in a well‐established prophylactic Brown Norway rat model. IgG1, IgE, and IgA responses, allergy elicitation, intestinal and mLN gene expression, and protein uptake are analyzed. The study demonstrates that camel and cow's milk in general has an insignificant cross‐preventive capacity. Yet, whereas cow's milk is shown to have a low transient capacity to prevent sensitization and clinically active camel milk allergy, camel milk does not show this effect for CMA.

**Conclusions:**

This study suggests that due to lack of cross‐tolerance camel milk cannot be used for CMA prevention.

## Graphical abstract

Camel milk has gained increasing interest as an alternative protein source for infant formula manufacture. In Brown Norway rats, we investigated the cow's milk allergy preventive capacity of camel milk and vice versa, and demonstrated that camel milk could not prevent cow's milk allergy, while cow's milk had a transient capacity to prevent camel milk allergy.

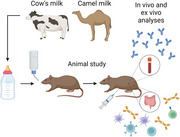

## 1 Introduction

Cow's milk is one of the most common sources of protein causing IgE‐mediated food allergy in infants and small children.^[^ ^1^ ^]^ Cow's milk allergy (CMA) affects 0.5–3.8% of small children, however, the prevalence varies between different countries.^[^ ^1^, ^2^ ^]^ For CMA management, strict avoidance of intact cow's milk proteins is needed, and for infants not exclusively breastfed the use of hypoallergenic infant formula is recommended.^[^ ^3^ ^]^ For CMA prevention in infants and small children at high‐risk of developing CMA, there are currently no specific recommendations, neither for the use of particular infant formulas if breastfeeding is not possible nor for when to introduce cow's milk proteins,^[^ ^4^, ^5^ ^]^ although delayed introduction of common allergens is discouraged.^[^ ^4^, ^5^ ^]^ Yet, in a study by Urashima et al.^[^ ^6^ ^]^ it was shown that avoidance of cow's milk based formula in the first days of life prevented the development of CMA. In contrast to current guidelines, previous guidelines recommended the use of partially hydrolyzed infant formula (pHF) for the prevention of CMA in infants not exclusively breastfed and at high‐risk of developing CMA,^[^ ^7^, ^8^ ^]^ but as current evidence from human studies point to no benefit of pHF in preventing CMA, this recommendation has been omitted.^[^ ^4^, ^9^, ^10^ ^]^

A few human studies have assessed the preventive capacity of pHF in infants at high‐risk of developing CMA, and while some showed the benefit of pHFs in preventing CMA when compared to conventional formulas,^[^ ^11^, ^12^, ^13^ ^]^ Lowe et al.^[^ ^14^ ^]^ reported no benefit. In line with the study of Lowe et al.,^[^ ^14^ ^]^ three systematic reviews stressed that there is no evidence for a preventive capacity of pHFs.^[^ ^15^, ^16^, ^17^ ^]^ Also animal studies showed different outcomes, where some studies demonstrated no differences in the capacity of intact and hydrolyzed cow's milk products in preventing sensitization to cow's milk proteins,^[^ ^18^, ^19^, ^20^, ^21^ ^]^ while another study showed that partially hydrolyzed whey proteins could only partly prevent CMA.^[^ ^22^ ^]^ Thus, based on human as well as animal studies it seems there is no unambiguous evidence for the benefit of using pHF in the prevention of CMA. Whereas pHFs have previously been recommended in the prevention of CMA, infant formulas based on extensively hydrolyzed cow's milk proteins and on amino acids are only recommended for CMA management.^[^ ^23^ ^]^ Further, pro‐ and prebiotics such as *Lactobacillus rhamnosus GG* and galacto‐/fructooligisaccharide, respectively, are known to have a positive impact on gut microbiota composition and thus suggested to accelerate development of tolerance towards cow's milk proteins.^[^ ^24^, ^25^ ^]^

There seems to be a growing interest in new types of infant formulas based on non‐hydrolyzed proteins, both as alternatives to conventional infant formulas but also for the prevention and management of CMA.^[^ ^5^ ^]^ For example, in a study by Graversen et al., investigating the impact of heat‐treatment on the allergy preventive capacity of whey proteins, it was shown that heat‐treated whey protein and the unmodified counterpart were equally good in inducing tolerance towards whey protein but that the heat‐treated version had a lower allergenicity.^[^ ^26^ ^]^

Infant formulas based on milk proteins from goat, sheep, donkey, horse, and camel^[^ ^27^, ^28^, ^29^, ^30^, ^31^, ^32^ ^]^ have gained an increasing interest as alternative protein sources to cow's milk protein.^[^ ^5^ ^]^ Especially, camel milk has received a growing interest in the recent years for its use in infant formulas, and in particular for its utility in CMA management.^[^ ^33^, ^34^, ^35^, ^36^ ^]^ This is mainly attributed to the low homology between camel and cow's milk proteins,^[^ ^29^, ^36^, ^37^ ^]^ as well as due to the lack of β‐lactoglobulin (BLG) in camel milk, which is one of the major allergens in cow's milk.^[^ ^38^ ^]^ In a recent animal study, we demonstrated that cow's and camel milk possessed similar inherent immunogenicity and allergenicity, but that the cross‐reactivity between counterpart proteins was low.^[^ ^34^ ^]^ In line, several studies analyzing blood samples from cow's milk allergic children showed no or low IgE binding to camel milk proteins confirming low cross‐reactivity between cow's and camel milk proteins.^[^ ^39^, ^40^, ^41^, ^42^ ^]^ Further, in human trials it has been shown that camel milk is well tolerated by the majority of cow's milk allergic children, stressing its potential in CMA management.^[^ ^29^, ^33^, ^36^ ^]^ However, whether or not early introduction of camel milk could drive a tolerance to cow's milk proteins has to our knowledge not been investigated. Therefore, in the present study, we assessed the capacity of cow's and camel milk to induce cross‐tolerance and investigated whether camel milk could prevent CMA and whether cow's milk could prevent camel milk allergy. For this purpose, a well‐established prophylactic Brown Norway (BN) rat model was used.^[^ ^19^, ^21^, ^26^ ^]^

## 2 Experimental Section

### 2.1 Milk Products

Cow's milk powder (MlekPol, Grajewo, Poland) was purchased in a local Polish shop, while camel milk powder was kindly provided by Ausnutria Dairy (China) Co., Ltd., (Changsha, Hunan, China). Solutions of the products were prepared by dissolving cow's and camel milk powders in sterile PBS (137 mM NaCl, 3 mM KCl, 8 mM Na_2_HPO_4_, 1 mM KH_2_PO_4_, pH 7.2) to a protein concentration of 50 mg mL^−1^ and stored at −20 °C until used. Endotoxin content was measured by Pierce LAL Chromogenic Endotoxin Quantitation Kit (88282, Thermo Fisher, Waltham, MA, USA) in accordance with the manufacturer's instruction. The endotoxin level of camel milk was <2 endotoxin units (EU) per mg protein, while the endotoxin level of cow's milk was approx. 9 EU per mg of protein.

### 2.2 SDS‐PAGE

In order to evaluate cow's and camel milk protein profiles, SDS‐PAGE was performed as previously described^[^ ^34^ ^]^ with minor changes. Briefly, cow's and camel milk proteins (15 µg) were separated under reducing conditions using a 4–20% precast polyacrylamide gel (Mini‐Protean TGX Stain‐Free gel, 4568094, Bio‐Rad, Hercules, CA, USA). Proteins were visualized by Bio Safe Coomassie (1610786, Bio‐Rad) and photographed using the Imager ChemiDoc XRS+ (Bio‐Rad).

### 2.3 Rats

BN rats from the in‐house breeding colony at the National Food Institute, Technical University of Denmark were kept in macrolon cages (*n* = 2 per cage) at 22 ± 1 °C with 55 ± 5% relative humidity and with a 12 h light–dark cycle. Rats were inspected once a day and weighted once a week. Rats were kept on an in‐house prepared diet free from milk proteins, with rice, fish, and potato as protein sources, for >10 generations. Diet and water were given ad libitum.

The animal experiment was carried out at the National Food Institute, Technical University of Denmark under ethical approval given by the Danish Animal Experiments Inspectorate and the authorization number 2020‐15‐0201‐00500‐C1. The experiment was overseen by the Technical University of Denmark's in‐house Animal Welfare Committee for animal care and use.

### 2.4 Dosage Regimen

**Figure 1:**
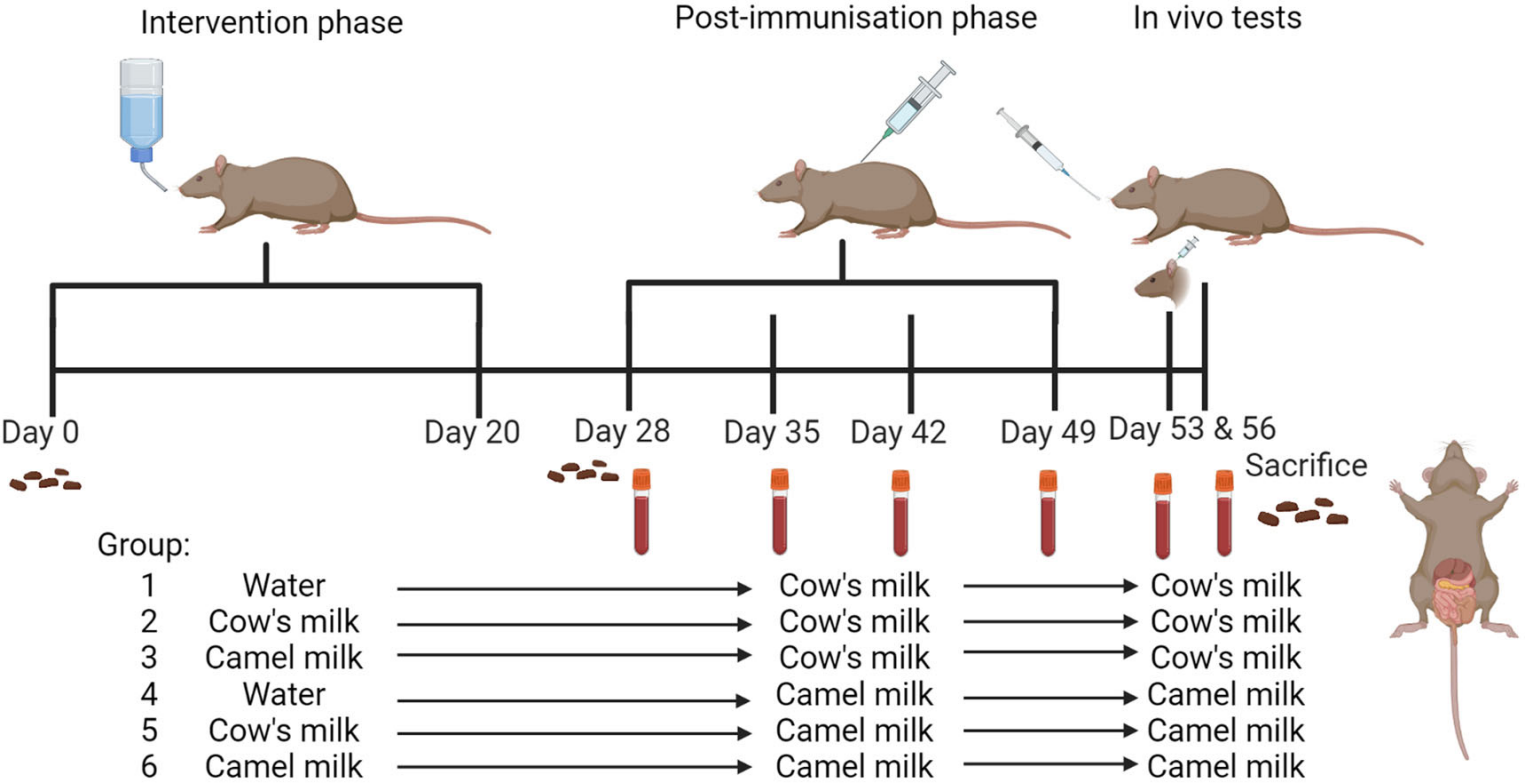
Outline of primary prevention animal experimental design. In the intervention phase (Day 0–20), Brown Norway (BN) rats were ad libitum administered with water as a control, cow's milk, or camel milk in their drinking bottles for 21 days. Subsequently, in the post‐immunization phase, the BN rats were intraperitoneally (i.p.) immunized with either cow's milk or camel milk for a total of four times at a 1‐week interval (Day 28, 35, 42, and 49). At Day 53 an ear swelling test and at Day 56 oral food challenge (OFC) were performed with either cow's milk or camel milk, corresponding to the post‐immunization and subsequently the BN rats were sacrificed. Blood samples were collected at Day 28, 35, 42, 49, 53, and 56, whereas fecal samples were collected at Day 0, 28, and 56. Samples of small intestine (SI) content, mesenteric lymph nodes (mLN), pieces of SI, lamina propria (LP), epithelium (EPI), and Peyer's patches (PP) were collected at the day of sacrifice (Day 56). Figure created with BioRender.com.

To evaluate the capacity of cow's and camel milk in preventing CMA and camel milk allergy, an animal experiment was performed. The animal experiment was divided into two phases; an intervention phase and a post‐immunization phase which was completed with in vivo tests, as displayed in **Figure** **1**. A total of 48 BN rats, 4–7 weeks of age, were divided into six groups of eight rats (*n* = 4 per gender). For investigating prevention of CMA, groups of rats (Group 1–3) received either water as a control, cow's milk, or camel milk ad libitum in their drinking bottles for 21 days (Intervention phase: Day 0–20) for the purpose of inducing oral tolerance (Figure 1). Similarly, for investigating prevention of camel milk allergy, groups of rats (Group 4–6) received either water as a control, cow's milk, or camel milk ad libitum in their drinking bottles for 21 days (Intervention phase: Day 0–20) for the purpose of oral tolerance induction (Figure 1). Milk protein concentration in drinking bottles was 12.5 g protein L^−1^. Subsequently, after 1 week of rest, rats were post‐immunized intraperitoneally (i.p.) with either 100 µg of cow's milk proteins (Group 1–3) or 100 µg of camel milk proteins (Group 4–6) in 0.5 mL PBS, once a week for 4 weeks (post‐immunization phase: Day 28, 35, 42, and 49). The animal experiment was completed with two in vivo tests, where an ear swelling test was performed at Day 53, and an oral food challenge (OFC) was performed at Day 56. Rats were sacrificed 1 week after last post‐immunization at Day 56 by exsanguination using carbon dioxide inhalation as anesthesia and blood were collected. Throughout the experiment, blood samples were collected from the sublingual vein; after the intervention phase at Day 28 and 1 week after each post‐immunization at Day 35, 42, 49, and 53. In addition, fecal samples were collected at Day 0, 28, and 56. At sacrifice (Day 56) the following samples were collected; small intestine (SI) content, mesenteric lymph nodes (mLN), pieces of SI, lamina propria (LP), epithelium (EPI), and Peyer's patches (PP). Blood samples were converted into serum, and fecal and SI content samples were converted into fecal and SI content water, respectively, as previously described.^[^ ^43^ ^]^

### 2.5 In Vivo Tests

At Day 53 an ear swelling test was performed as previously described.^[^ ^44^ ^]^ Briefly, rats were anesthetized with hypnorm/midazolam and the initial ear thickness was measured. Subsequently, rats were intradermally (i.d.) injected with 20 µL of PBS with either 10 µg of cow's milk protein (Group 1–3) or 10 µg of camel milk protein (Group 4–6) into one ear. Ear thickness was measured again 15 min after injection, and delta ear swelling was calculated for each animal. Further, at Day 56, an OFC was performed, where rats from group 1–3 were intragastrically (i.g.) challenged with 1 mL of PBS with 100 mg of cow's milk protein and rats from group 4–6 were i.g. challenged with 1 mL of PBS with 100 mg of camel milk protein. Rats were observed for 10 min in order to monitor number of upper gastro‐intestinal symptoms by means of singultus‐ and emesis‐like behavior, indicating the experience of nausea.

### 2.6 Indirect ELISA for Specific IgG1 Detection

To detect serum IgG1 specific for either cow's or camel milk, indirect ELISAs were performed, as previously described.^[^ ^34^ ^]^ Results were expressed as log2 titre values and defined as the interpolated dilution of the given serum sample leading to the mean absorbance for the negative control +3 SD.

### 2.7 Indirect ELISA for Specific IgA Detection

To detect serum IgA specific for either cow's milk or camel milk, indirect ELISAs were performed. Maxisorp microtitre plates (96‐well, Nunc, Roskilde, Denmark) were coated with 100 µL/well of 10 µg mL^−1^ of cow's milk or camel milk in coating buffer (15 mM Na_2_CO_3_, 35 mM NaHCO_3_, pH 9.6), and incubated overnight at 4 °C. Plates were washed five times between each step in PBS with 0.01% w/v Tween 20 (P1379, Sigma‐Aldrich) (PBS‐T). For all steps that required incubation, plates were incubated for 1 h in the dark at RT with gentle agitation. First, plates were incubated with 50 µL per well of twofold serial dilutions of serum samples v/v in PBS‐T. In each plate, positive and negative control sera were included in order to identify potential plate‐to‐plate variance. For IgA detection, 50 µL per well of HRP‐labeled goat‐anti‐rat IgA (STAR 111P, Bio‐Rad) diluted 1:5000 (v/v) in PBS‐T was added to the plates. Next, plates were additionally washed twice with tap water. To visualize the detection of specific IgA, 100 µL per well of TMB‐one (3,3’,5,5’‐tetramethylobenzidine, 4380A, Kementec Diagnosis, Taastrup, Denmark) was added and incubated for 12 min at RT. The reaction was stopped by adding 100 µL per well of 0.2 M H_2_SO_4_, and the absorbance was measured at 450 nm with a reference wavelength of 630 nm using a microtitre reader (Gen5, BioTek, EL800 Instrument, Winooski, VT, USA). Results were expressed as log2 titre values and defined as the interpolated dilution of the given serum sample leading to the mean absorbance for the negative control +3 SD.

### 2.8 Sandwich ELISA for Total IgA Detection

To detect total IgA in serum, fecal water, and SI content water, sandwich ELISA was performed as previously described.^[^ ^28^ ^]^ Results were expressed as log2 titre values and defined as the interpolated dilution of the given serum sample leading to the mean absorbance for the negative control +3 SD.

### 2.9 Antibody‐Capture ELISA for Specific IgE Detection

To detect serum IgE specific for cow's or camel milk, antibody‐capture ELISAs were performed as previously described,^[^ ^34^ ^]^ except of 5% v/v horse serum (S0910‐500, Biowest, Nuaille, France) being used as blocking agent. Further, for both products, serum samples were twofold diluted in 5% v/v horse serum. DIG‐coupled cow's or camel milk proteins were used with a final concentration of 0.03 µg mL^−1^ in PBS‐T for specific IgE detection. Results were expressed as the log2 titre values and defined as the interpolated dilution of the given serum sample leading to the mean absorbance for the negative control +3 SD.

### 2.10 Immunoblotting

To detect immune reactive proteins, immunoblotting with serum pools from each group of rats was performed. In brief, SDS‐PAGE with 5 µg of cow's and camel milk proteins were performed, and proteins transferred onto polyvinylidene difluoride (PVDF) membranes as previously described.^[^ ^34^ ^]^ Serum pools from the day of sacrifice (Day 56) were diluted 1:3000 v/v, whereas the secondary antibody was diluted 1:15,000 and added together with StrepTacin‐HRP conjugate (Bio‐Rad) diluted 1:20,000 for protein standard visualization. All membranes were developed for 15 s for optimal visualization and direct comparison.

### 2.11 Tissue RNA Extraction, cDNA Synthesis, and RT‐qPCR

To evaluate expression of different genes of interest, mLN and 2 × 0.5 cm pieces of SI, harvested 27 and 37 cm distal from the stomach were collected and stored in RNAlater (Invitrogen, Carlsbad, CA, USA) at −20 °C. RNA extraction, cDNA synthesis, and RT‐qPCR were performed as previously described.^[^ ^43^ ^]^ For RNA extraction from mLN, QIAzol Lysis Reagent (79306, Qiagen, Hilden, Germany) and RNeasy Lipid Tissue Mini Kit (74804, Qiagen) were used in accordance with manufacturers’ protocol. Taqman gene assays (Applied Biosystems, Thermo Fisher Scientific, MA, USA) used were: Ocln (occludin Rn00580064_m1), TSLP (thymic stromal lymphopoietin, Rn01761072_m1), IL‐1β (interleukin 1beta, Rn00580432_m1), IL‐4 (interleukin 4, Rn01456866_m1), IL‐10 (interleukin 10, Rn01483989_m1), INF‐γ (interferon gamma, Rn00594078_m1), TGF‐β (transforming growth factor beta, Rn005720_m1), FoxP3 (forkhead box P3, Rn01525092_m1), and CX3XR1 (C‐X3‐C chemokine receptor 1, Rn00591798_m1). The levels of different gene expression were shown as the relative gene expression by means of 2^−deltaCT^ method using B2m (Beta‐2‐microglobulin Rn00560865_m1) and Sdha (Succinate dehydrogenase complex Rn00590475_m1) as normalization genes.

### 2.12 In Vivo Intestinal Protein Uptake

To evaluate protein uptake in the SI compartments, LP, SI content, PP, and EPI of BN rats challenged with cow's milk (Group 1–3) were harvested and homogenized as previously describe.^[^ ^26^ ^]^ BLG ELISA kit (Bethyl Laboratories, Montgomery, TX, USA) was used for determination of concentration of BLG, as a marker for cow's milk protein uptake, in supernatants prepared from tissue homogenates in accordance to manufacturers’ protocol, as previously described.^[^ ^26^ ^]^

### 2.13 Statistical Analysis

Graphs and statistical analyses were made using GraphPad Prism version 9.0.1 (San Diego, CA, USA). Results from ELISAs were expressed as log2 antibody titre values. All data were initially tested for normal distribution using D'Agostino‐Pearson normality test. If the data passed normality test, differences between two groups were analyzed using parametric *t‐*test while differences between three or more groups were analyzed using one‐way ANOVA followed by Bonferroni post‐test. If the data did not pass normality test, differences between two groups were analyzed using non‐parametric Mann–Whitney *U* test while differences between three or more groups were analyzed using Kruskal–Wallis test followed by Dunn's post‐test for multiple comparison. Differences were significant if *p* ≤ 0.05. Asterisk(s) indicate statistically significant differences between groups: *p ≤ 0.05, **p ≤ 0.01, ***p ≤ 0.001, ****p ≤ 0.0001.

## 3 Results

### 3.1 Humoral Immune Responses

**Figure 2:**
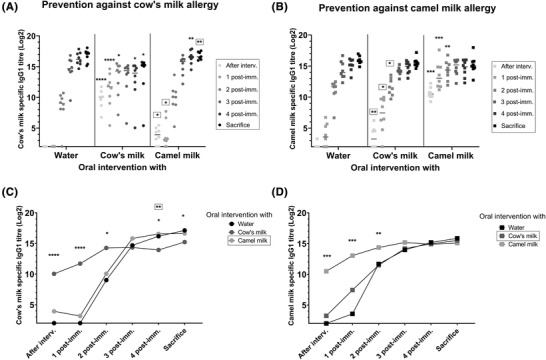
Specific IgG1 responses. A) IgG1 specific for cow's milk in groups intervened with either water, cow's milk, or camel milk and tested for prevention against cow's milk allergy. B) IgG1 specific for camel milk in groups intervened with either water, cow's milk, or camel milk and tested for prevention against camel milk allergy. A, B) Each symbol represents individual rats at different time points of the experiment, and horizontal lines indicate median values. C) Progression of the cow's milk‐specific immune responses after oral interventions with either: ● water,   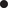 cow's milk, or   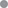 camel milk and subsequent post‐immunizations with cow's milk. D) Progression of the camel milk‐specific immune responses after oral interventions with either: ■ water,   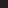 cow's milk, or   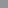 camel milk and subsequent post‐immunizations with camel milk. C, D) Each symbol represents median value of different groups at different time points of the experiment. Kruskal–Wallis test followed by Dunn's post‐test was applied, where statistically significant differences to the water intervened group (unframed) and relative to A) cow's milk or B) camel milk intervened group (framed), or between C) cow's milk or D) camel milk and the water intervened group (unframed), and between C) camel milk and D) cow's milk and the water intervened group (framed) are shown as *p ≤ 0.05, **p ≤ 0.01, ***p ≤ 0.001, ****p ≤ 0.0001. Interv., intervention; post‐imm., post‐immunization.

To evaluate the humoral immune responses developed against cow's or camel milk proteins throughout the study, levels of specific IgG1 were measured at six different time points. At first, it was shown that ad libitum bottle administration of both cow's and camel milk promoted the development of specific IgG1 during the intervention phase, as rats administered with cow's milk developed a statistically significant cow's milk‐specific IgG1 response and rats administered with camel milk developed a statistically significant camel milk‐specific IgG1 response, when comparing with water administered rats (**Figure** **2**A,B). A statistically significant lower cross‐reactive immune response was observed after the intervention phase for rats intervened with camel milk and tested against cow's milk when compared to cow's milk intervened rats, though most camel milk intervened rats had IgG1 that could also react with cow's milk (Figure 2A). Similarly, a statistically significant lower cross‐reactive immune response was observed for rats intervened with cow's milk and tested against camel milk when compared to camel milk intervened rats, where fewer cow's milk intervened rats had IgG1 that could also react with camel milk (Figure 2B).

Subsequently, the progression of the immune responses upon each of the post‐immunizations with either cow's milk or camel milk was assessed, demonstrating that levels of IgG1 specific for cow's milk (Figure 2A,C) or camel milk (Figure 2B,D) increased until reaching a plateau after the second or third post‐immunization. Yet, for rats post‐immunized with cow's milk, the cow's milk‐specific IgG1 response was statistically significant lower in rats intervened with cow's milk compared to rats intervened with either water or camel milk at sacrifice (Figure 2A). In contrast, such differences were not seen between groups of rats post‐immunized with camel milk (Figure 2B).

### 3.2 Prevention of Sensitization

**Figure 3:**
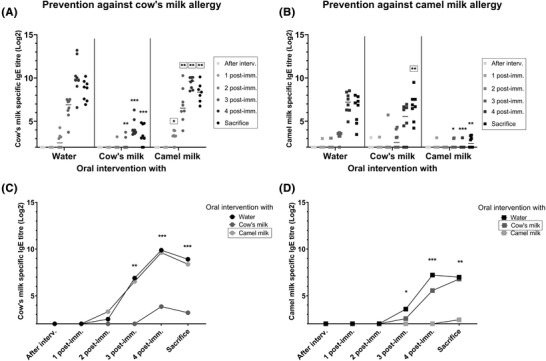
Specific IgE responses. A) IgE specific for cow's milk in groups intervened with either water, cow's milk, or camel milk and tested for prevention against cow's milk allergy. B) IgE specific for camel milk in groups intervened with either water, cow's milk, or camel milk and tested for prevention against camel milk allergy. A, B) Each symbol represents individual rats at different time points of the experiment, and horizontal lines indicate median values. C) Progression of the cow's milk‐specific IgE responses after oral interventions with: ● water,    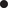 cow's milk, or   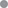 camel milk and subsequent post‐immunizations with cow's milk. D) Progression of the camel milk‐specific IgE responses after oral interventions with either: ■ water,   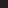 cow's milk, or   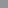 camel milk and subsequent post‐immunizations with camel milk. C, D) Each symbol represents median value of different groups at different time points of the experiment. Kruskal–Wallis test followed by Dunn's post‐test was applied, where statistically significant differences to the water intervened group (unframed) and relative to A) cow's milk or B) camel milk intervened group (framed), or between C) cow's milk or D) camel milk and water intervened group (unframed) are shown as *p ≤ 0.05, **p ≤ 0.01, ***p ≤ 0.001, ****p ≤ 0.0001. Interv., intervention; post‐imm., post‐immunization.

To evaluate the capacity of cow's and camel milk in preventing sensitization to both cow's and camel milk, levels of specific IgE were measured throughout the animal experiment. Neither ad libitum bottle administration with cow's nor camel milk induced sensitization in the intervention phase, as specific IgE could not be detected in any of the groups (**Figure** **3**A,B). During the post‐immunization regime it was revealed that cow's milk was the most efficient in preventing cow's milk sensitization (Figure 3A,C) and camel milk was the most efficient in preventing camel milk sensitization (Figure 3B,D). In fact, whereas cow's milk was efficient in preventing sensitization towards cow's milk to a statistically significant degree after the third and fourth post‐immunization, camel milk did not show any capacity to prevent sensitization to cow's milk, as at no time point was camel milk capable of inhibiting the development of cow's milk specific IgE when compared to intervention with water (Figure 3A,C). Similarly, camel milk was efficient in preventing camel milk sensitization to a statistically significant degree after the third and fourth post‐immunization, whereas cow's milk seemed only to have a low capacity to prevent sensitization toward camel milk (Figure 3B,D). Yet, in the cow's milk intervened rats a transient reduction in the camel milk‐specific IgE level compared to the water intervened rats was observed after the fourth post‐immunizations (*p* = 0.0482, Mann–Whitney *U* test between cow's milk and water intervened groups).

### 3.3 Prevention of Clinical Responses

**Figure 4:**
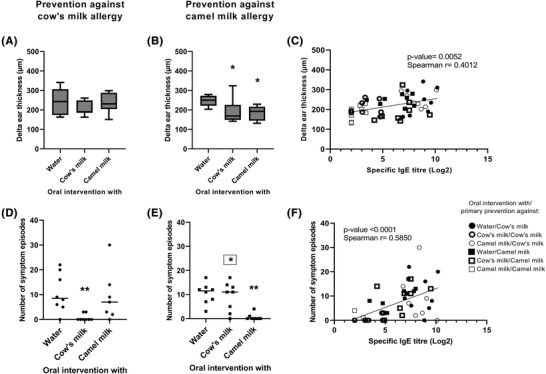
Clinical responses. A) Ear swelling test with cow's milk in groups intervened with either water, cow's milk, or camel milk and tested for prevention against cow's milk allergy. B) Ear swelling test with camel milk in groups intervened with either water, cow's milk, or camel milk and tested for prevention against camel milk allergy. C) Correlation between IgE specific for the product rats were post‐immunized with and delta ear thickness after intradermal injection with the same product. D) Symptom episodes after oral food challenge (OFC) with cow's milk in groups intervened with either water, cow's milk, or camel milk and tested for prevention against cow's milk allergy. E) Symptom episodes after OFC with camel milk in groups intervened with either water, cow's milk, or camel milk and tested for prevention against camel milk allergy. F) Correlation between IgE specific for the product rats was post‐immunized with and number of symptom episodes after OFC with the same product. C–F) Each symbol represents a single rat, and A, B, D, E) horizontal lines indicate median value. A, B, D, E) Kruskal–Wallis test followed by Dunn's post‐test was applied. Statistically significant differences to the water intervened group (unframed) A, B, D, E) and relative to cow's milk intervened group A, C) or camel milk intervened group B, D) (framed) are shown as *p ≤ 0.05, **p ≤ 0.01. Non‐parametric Spearman correlations were calculated between all pairs of specific IgE titre and delta ear thickness or number of symptom episodes C, F).

To evaluate the capacity of cow's and camel milk in preventing clinical responses towards cow's and camel milk, an ear swelling test and an OFC were performed. In the ear swelling test, groups post‐immunized with cow's milk, and hence tested for prevention of CMA, showed no statistically significant differences in ear swelling after i.d. injection of cow's milk regardless of the oral intervention (**Figure** **4**A). However, the group intervened with cow's milk showed a slightly lower but non‐significant ear swelling when compared with groups intervened with either water or camel milk. Contrary, groups post‐immunized with camel milk, and hence tested for prevention of camel milk allergy, showed statistically significant differences in their ear swelling responses. A significantly lower ear swelling after i.d. injection of camel milk was observed for groups intervened with camel milk and cow's milk when compared to the group intervened with water (Figure 4B). These results were aligned with the specific IgE results, and a statistically significant positive correlation between specific IgE levels and delta ear thickness was observed, stressing the functional relevance of the specific IgE raised in the rats (Figure 4C).

The number of symptom episodes was counted after OFC with the product the rats were post‐immunized with. The number of symptom episodes after OFC with cow's milk was statistically significant lower for the group intervened with cow's milk in comparison to the groups intervened with either water or camel milk (Figure 4D). Similarly, the number of symptom episodes after OFC with camel milk was statistically significant lower for the group intervened with camel milk in comparison to the groups intervened with either water or cow's milk (Figure 4E). These results were also clearly aligned with the specific IgE results, where a statistically significant positive correlation was observed between specific IgE and number of symptom episodes (Figure 4F), confirming that levels of specific IgE were indicative for the clinical relevance of the allergy. Thus, the results demonstrated that neither could camel milk prevent sensitization nor clinical reactions against cow's milk, whereas cow's milk had a low capacity to prevent sensitization and clinical reactions against camel milk.

### 3.4 IgG1 Milk Protein Specificity

In order to evaluate the specificity of IgG1 and the cow's and camel milk protein binding profiles, SDS‐PAGE followed by immunoblotting was performed.

**Figure 5:**
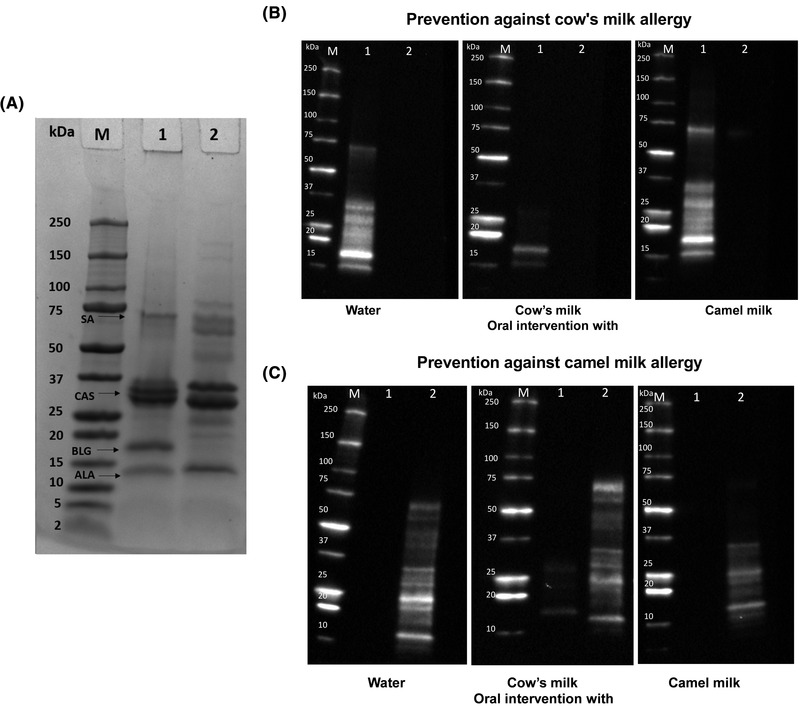
Specificity of IgG1 responses. A) SDS‐PAGE for visualization of cow's (lane 1) and camel milk (lane 2) protein profiles. B) Immunoblotting to visualize specificity of IgG1 responses towards cow's milk (lane 1) and camel milk (lane 2) in rats intervened with either water, cow's milk, or camel milk and tested for prevention against cow's milk allergy. C) Immunoblotting to visualize specificity of IgG1 responses towards cow's milk (lane 1) and camel milk (lane 2) in rats intervened with either water, cow's milk, or camel milk and tested for prevention against camel milk allergy. ALA, α‐lactalbumin; BLG, β‐lactoglobulin; CAS, caseins; kDa, kilodalton; M, molecular weight standard marker; SA, serum albumin.

For both cow's and camel milk, caseins were shown as thick bands between 25 and 37 kDa (**Figure** **5**A). In addition, α‐lactalbumin (ALA) was shown for both products as a band corresponding to the molecular weight of approx. 15 kDa. A thick band between 15 and 20 kDa was only visible for cow's milk, representing BLG, which is not present in camel milk. Finally, both cow's and camel milk had a band visible just below 75 kDa, corresponding to serum albumin (SA).

The reactivity of IgG1 for rats post‐immunized with cow's milk and hence tested for prevention against CMA was lower for the group intervened with cow's milk compared to that of the water and camel milk intervened rats (Figure 5B). Bands around 15 kDa representing reactivity with ALA and between 15 and 20 kDa representing reactivity with BLG, were the only bands detected and with a clearly lower intensity for rats intervened with cow's milk compared to rats intervened with either water or camel milk that in contrast showed reactivity towards all major cow's milk proteins (Figure 5B), indicating that the humoral immune response towards SA and caseins was more readily restrained compared to ALA and BLG.

Similarly, the reactivity of IgG1 for rats post‐immunised with camel milk and hence tested for prevention against camel milk was slightly lower for the group intervened with camel compared to water and cow's milk intervened rats (Figure 5C). Bands around 15 kDa representing reactivity with ALA and between 25 and 37 kDa representing reactivity with caseins, were the bands detected and with a lower intensity for rats intervened with camel milk compared to rats intervened with water or cow's milk that in contrast showed reactivity towards all major camel milk proteins (Figure 5C). Interestingly, a weak band around 15 kDa was visible in cow's milk intervened rats but not in water or camel milk intervened rats, indicating that a solid IgG1 response specific for cow's milk ALA was raised already in the intervention phase (Figure 5C).

### 3.5 Intestinal Immune Responses

**Figure 6:**
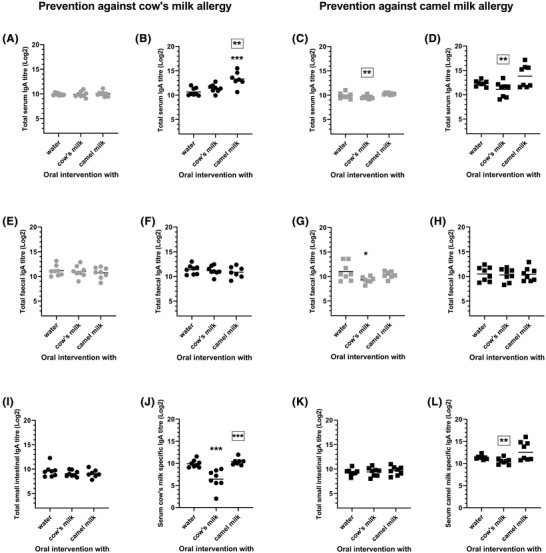
Total and specific IgA. Total IgA in serum A) after intervention phase or B) at sacrifice from rats intervened with either water, cow's milk, or camel milk and tested for prevention against cow's milk allergy. Total IgA in serum C) after intervention phase or D) at sacrifice from rats intervened with either water, cow's milk, or camel milk and tested for prevention against camel milk allergy. Total IgA in feces E) after intervention phase or F) at sacrifice from rats intervened with either water, cow's milk, or camel milk and tested for prevention against cow's milk allergy. Total IgA in feces G) after intervention phase or H) at sacrifice from rats intervened with either water, cow's milk, or camel milk and tested for prevention against camel milk allergy. I) Total IgA in small intestine content from rats intervened with either water, cow's milk, or camel milk and tested for prevention against cow's milk allergy. J) Serum cow's milk‐specific IgA from rats intervened with either water, cow's milk, or camel milk and tested for prevention against cow's milk allergy. K) Total IgA in small intestine content from rats intervened with either water, cow's milk, or camel milk and tested for prevention against camel milk allergy. L) Serum camel milk‐specific IgA from rats intervened with either water, cow's milk, or camel milk and tested for prevention against camel milk allergy. Each symbol represents individual rats and horizontal lines indicate mean value. Each color represents different time points of the experiment: (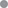), (■) represents results after intervention phase while (●), (■) represents results at the day of sacrifice. One‐way ANOVA followed by Bonferroni post‐test was applied to the water intervened group (unframed) and relative to cow's milk intervened group (A, B, E, F, I, J) or to camel milk intervened group (C, D, G, H, K, L) (framed). Statistically significant differences are shown as *p ≤ 0.05, **p ≤ 0.01, ***p ≤ 0.001.

The level of total IgA was determined in serum, feces and SI content. In general, for all groups of rats, the total IgA in serum increased throughout the study and was statistically significant higher at sacrifice compared to after the intervention phase (Figure S1A,B, Supporting Information), indicating a general impact of either age or the post‐immunizations on the total IgA level in serum. There were no statistically significant differences between total IgA levels in serum after the intervention phase between the water and cow's or camel milk intervened rats (**Figure** **6**A,C). Contrary, a higher (*p* = 0.0004 and 0.1466, based on one‐way ANOVA) total IgA level was observed for rats intervened with camel milk compared to rats intervened with water and post‐immunized with cow's and camel milk, respectively, indicating an effect of the camel milk intervention revealed upon post‐immunizations (Figure 6B,D). Further, rats intervened with camel milk had a statistically significant higher level of IgA than cow's milk intervened rats, irrespective of the post‐immunization (Figure 6B,D).

In line with the total IgA in serum, in general total IgA in feces increased throughout the study, even though statistically significant differences were only observed between naïve rats and at the day of sacrifice for rats intervened with cow's milk and post‐immunized with cow's milk and for rats intervened with camel milk and post‐immunized with camel milk (Figure S1C,D, Supporting Information). No differences in total IgA in feces were observed between rats intervened with water and rats intervened with cow's or camel milk at any time point irrespectively of the post‐immunization (Figure 6E–H) except from rats intervened with cow's milk and post‐immunized with camel milk (Figure 6G).

Total IgA in SI content at the day of sacrifice did not show significant differences between water, cow's and camel milk intervened rats regardless the post‐immunization (Figure 6I,K), suggesting that neither intervention nor post‐immunization influenced gut mucosal immune homeostasis by changes in IgA.

For rats tested for prevention against CMA, intervention with cow's milk promoted a statistically significant lower level of cow's milk‐specific IgA in serum in comparison to rats intervened with water and camel milk (Figure 6J). In contrast, for rats tested for prevention against camel milk allergy, intervention with camel milk did not promote a lower level of camel milk‐specific IgA in serum compared to intervention with water or cow's milk, but instead intervention with cow's induced a statistically significantly lower level of camel milk‐specific IgA in serum in comparison to rats intervened with camel milk (Figure 6H). This indicates that intervention with cow's milk in general promoted reduced levels of specific IgA.

### 3.6 Cellular Responses

**Figure 7:**
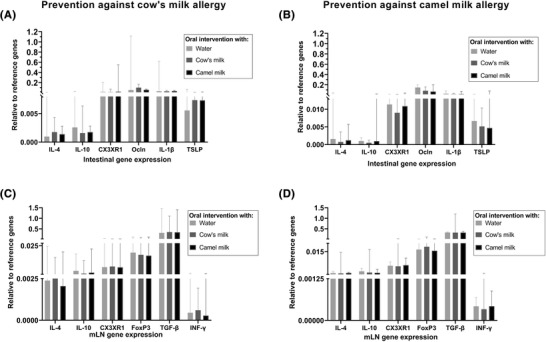
Relative gene expression. Expression of selected genes in the small intestine (SI) from rats intervened with either water, cow's milk, or camel milk and tested for prevention against A) cow's milk allergy or B) camel milk allergy. Expression of selected genes in mesenteric lymph nodes (mLN) from rats intervened with either water, cow's milk, or camel milk and tested for prevention against C) cow's milk allergy or D) camel milk allergy. Each bar represents the median with 95% confidence interval. Kruskal–Wallis test followed by Dunn's post‐test was applied, where statistical analysis was performed to the water intervened rats and relative to cow's milk A, C) or camel milk B, D) intervened group.

Cellular immune responses were evaluated by means of gene expression of the Th2 cytokine IL‐4, the tolerogenic biomarkers IL‐10, CX3XR1, FoxP3, TGF‐β, and IFN‐γ, the tight junction protein Ocln, the epithelial‐derived cytokine TSLP and the pro‐inflammatory cytokine IL‐1β in the SI and mLN. In general, no statistically significant differences were observed between any of the intervention groups regardless of whether rats were tested for prevention against cow's or camel milk allergy (**Figure** **7**A–D). Yet, for rats intervened with cow's milk and post‐immunized with camel milk a slightly lower expression of CX3XR1 and IL‐4 were observed when comparing with water intervened rats (*p* = 0.025 and 0.0289, respectively, based on Mann–Whitney *U* test, Figure 7B).

### 3.7 Intestinal Protein Uptake

**Figure 8:**
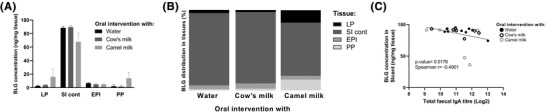
In vivo intestinal protein uptake in rats prevented against cow's milk allergy. A) β‐lactoglobulin (BLG) concentration in ng mg^−1^ of tissue. Each bar represents mean value with the standard error for the mean. Kruskal–Wallis test followed by Dunn's post‐test was applied. B) Relative distribution of BLG (%) between lamina propria (LP), small intestine content (SI cont), epithelium (EPI), and Peyer's patches (PP) compartments in rats intervened with either water, cow's milk, or camel milk and tested for prevention against cow's milk allergy. C) Correlation between total fecal IgA and amount of BLG in SI cont. Non‐parametric Spearman correlations were calculated between all pairs of total fecal IgA and amount of BLG in SI cont.

To evaluate intestinal protein uptake in rats intervened with water, cow's and camel milk and tested for prevention against CMA, and thus challenged with cow's milk at the end of the experiment, concentration of BLG as a marker of cow's milk protein uptake, was measured in LP, SI content, EPI, and PP. In general, no statistically significant differences were observed in the amount of BLG taken up in each individual intestinal compartment (**Figure** **8**A), even though it was observed that the overall distribution of BLG in the camel milk intervened group differed from the water and cow's milk intervened groups, with a higher proportion of BLG found in LP and PP, and with a consequential lower proportion in SI content (Figure 8B). Interestingly, a small but statistically significant inverse correlation between the amount of BLG in the SI content and total fecal IgA level was observed across all groups, suggesting that the more IgA secreted into the intestinal lumen the less BLG was trapped in the SI content (Figure 8C).

## 4 Discussion

At present, there are no specific recommendations for the use of any particular infant formula, including for or against the use of infant formulas based on hydrolyzed cow's milk proteins in the prevention of CMA.^[^ ^4^ ^]^ When breastfeeding is insufficient or impossible, various substitutes are available, but the choice of infant formula should be based on the need of each individual infant.^[^ ^4^, ^7^, ^9^, ^10^ ^]^ Hence, there could be an opportunity for new and alternative infant formulas.

When designing infant formulas for CMA prevention, it is the delicate balance between inducing a solid oral tolerance still avoiding the development of CMA that should be in focus. Yet, there is limited knowledge on whether the complete repertoire of allergens and epitopes is required to induce a solid tolerance, as previously discussed in several studies investigating the tolerance inducing capacity of hydrolysed cow's milk proteins.^[^ ^21^, ^45^, ^46^, ^47^, ^48^ ^]^ Not only may the initial epitope repertoire be different between intact and hydrolyzed cow's milk proteins, but peptide fragments may in addition be processed and presented to the gut immune system in a distinct way than their intact counterparts.^[^ ^45^ ^]^ Consequently, different repertoires of B and T cell epitopes may be presented to the immune system. In line, the same aspect and the possibility of bystander effects have also been discussed for allergy immunotherapy,^[^ ^49^ ^]^ where peptide‐based immunotherapy was shown to be effective in some studies,^[^ ^50^, ^51^ ^]^ whereas in other studies it was shown to lack tolerance inducing capacity.^[^ ^52^, ^53^ ^]^ Thus, it could be speculated that infant formulas based on intact proteins would be more efficient in preventing CMA than those based on hydrolyzed proteins as intact proteins may cover a larger repertoire of epitopes, containing both linear and conformational epitopes. Yet, this could be on the expense of their safety.

In the last decade, there has been a growing interest in the usability of milk proteins from other mammalian species for infant formulas.^[^ ^5^ ^]^ Whereas goat and sheep milk have been shown to cause allergic reactions in cow's milk allergic patients, probably due to the high homology of goat and sheep milk proteins with cow's milk proteins,^[^ ^41^, ^54^, ^55^ ^]^ donkey, horse, and camel milk have in general been shown to be a safer option for cow's milk allergic patients, probably due to the lower homology with cow's milk proteins.^[^ ^28^, ^29^, ^41^, ^56^ ^]^ Yet, none of them have been investigated for their CMA prevention potential. In this study, we focused on camel milk and evaluated whether it could prevent CMA and whether cow's milk could prevent camel milk allergy using a well‐established prophylactic BN rat model.^[^ ^19^, ^21^, ^26^ ^]^

We demonstrated that neither camel milk nor cow's milk induced sensitization upon 3 weeks of ad libitum administration. Yet, both camel and cow's milk were shown to induce specific IgG1 upon 3 weeks of ad libitum administration, indicating that they were easily recognized by the immune system, which is a prerequisite for oral tolerance induction.^[^ ^26^ ^]^ Moreover, in line with human studies,^[^ ^34^, ^39^, ^40^, ^41^, ^42^ ^]^ as well as in our previous i.p. sensitization study,^[^ ^34^ ^]^ a low cross‐reactivity between cow's and camel milk proteins was revealed.

Results showed that whereas cow's milk was efficient in preventing cow's milk sensitization, camel milk was not capable of preventing sensitization to cow's milk. Similarly, camel milk was efficient in preventing camel milk sensitization. Interesting, cow's milk was shown to have a small though transient capacity to prevent sensitization to camel milk. Similar patterns were demonstrated when evaluating the clinically relevance of the sensitization to cow's and camel milk, where cow's milk could prevent a clinically relevant CMA, and camel milk could easily prevent a clinically relevant camel milk allergy. Contrary, camel milk could not prevent clinical symptoms of CMA whereas cow's milk had a small effect on the clinical manifestation of camel milk allergy. These results were well aligned with immunoblotting results, showing no differences in the pattern of the presence and intensity of IgG1 reactive proteins between groups intervened with water and groups intervened with cow's and camel milk, when prevention was tested against camel and CMA, respectively.

Overall, our results showed a low cross‐tolerogenic capacity between cow's and camel milk proteins, probably due to their low protein homology, where protein sequence alignments demonstrate protein identities of 47–81%.^[^ ^34^ ^]^ Thus, the overlapping epitope repertoire between cow's and camel milk proteins seemed too low to provide solid cross‐prevention, and hence this study did not provide evidence for a bystander effect of co‐existing epitopes. Collectively, this stresses that camel milk would not be a suitable protein source for infant formulas for CMA prevention. We hypothesize that donkey and horse milk proteins due to even lower protein homologies with cow's milk proteins, with protein sequence identities of 46–74%^[^ ^5^ ^]^ would likewise not provide suitable sources for CMA prevention, whereas goat and sheep milk proteins, with protein sequence identifies to cow's milk proteins of 85–95%^[^ ^5^ ^]^ would probably be better options. It has been suggested that a high degree of homology is needed between proteins in order to obtain a bystander effect and drive tolerance towards counterpart proteins.^[^ ^57^ ^]^ For example, in several studies it was shown that birch pollen immunotherapy only had limited effect on the related apple allergy,^[^ ^58^, ^59^, ^60^ ^]^ due to too low homology between the main allergens in birch pollen and apple, i.e., Bet v 1 and Mal d 1.^[^ ^61^ ^]^ Contrary, a study by Elizur et al., reported that cashew oral immunotherapy was not only efficient in inducing tolerance to cashew, but also to pistachio,^[^ ^62^ ^]^ probably due to a general higher protein sequence homology between major allergens.^[^ ^63^ ^]^

Even though it was low and transient, this study demonstrated some cross‐tolerance inducing capacity of cow's milk against camel milk allergy. We hypothesize that the reason for cow's milk containing some cross‐preventive capacity in contrast to camel milk, is due to the fact that cow's milk contains all major milk allergens,^[^ ^64^ ^]^ whereas camel milk do not contain BLG. Consequently, camel milk may not be capable of inducing tolerance against BLG, which is one of the major allergens found in cow's milk for which it is reported that up to 80% of cow's milk allergic patients have specific IgE against.^[^ ^65^ ^]^ In contrast to camel milk, milk from goat, sheep, donkey, and horse contain BLG,^[^ ^66^ ^]^ and thus may be able to drive tolerance towards all cow's milk allergens.

Cow's and camel milk were both shown to have some immunomodulatory effects, yet they were shown to be distinct and seemed to be independent of their allergy preventive capacity. While cow's milk seemed to restrain immune responses, reflected in lower specific IgA levels and a slightly lower expression of intestinal IL‐4 and CX3CR1, camel milk seemed to have an immune stimulatory capacity, as camel milk promoted total serum IgA. Strong immunomodulatory properties of camel milk has also previously been suggested.^[^ ^67^ ^]^

It is yet important to highlight that results from animal studies may not necessarily translate directly to the human situation. For example, in present study, rats were not administered cow's or camel milk until after weaning, hence rats were not introduced to the infant formulas shortly after birth but rather at the time of complementary food ingestion,^[^ ^68^ ^]^ which may have affected the outcome of the study as e.g. the gut immune system of the rats were probably more mature than that of infants.

In conclusion, this study demonstrated a low cross‐tolerogenic capacity of camel and cow's milk proteins, indicating that camel milk is not a good candidate as a protein source for infant formulas in CMA prevention.

## Conflict of Interest

The study was performed during the PhD project of NZM, which was supported by Ausnutria Dairy (China) Co., Ltd, Changsha, Hunan, China. The company however did not participate in the study design, data acquisition, data analysis, interpretation, manuscript preparation, or the decision where to publish.

## Author Contributions

Conceptualization: N.Z.M. and K.L.B. Investigation and data curation: N.Z.M., M.H.S., and K.L.B. Methodology: N.Z.M., M.H.S., A.S.R.B., A.I.S., and K.L.B. Data visualization: N.Z.M., M.H.S., and K.L.B. Formal analysis: N.Z.M., M.H.S., and K.L.B. Supervision: K.L.B. Writing – original draft: N.Z.M. Writing – review & editing: N.Z.M., M.H.S., A.S.R.B., A.I.S., E.B.H., and K.L.B. All authors made substantial intellectual contributions to the study, reviewed the manuscript critically, and approved the final version of the manuscript.

## Supporting information

Supporting Information

## Data Availability

The data that support the findings of this study are available from the corresponding author upon reasonable request.

## References

[1] J. D. Flom , S. H. Sicherer , Nutrients 2019, 11, 10.3390/nu11051051 PMC6566637 31083388

[2] B. Zepeda‐Ortega , A. Goh , P. Xepapadaki , A. Sprikkelman , N. Nicolaou , R. E. H. Hernandez , A. H. A. Latiff , M. T. Yat , M. Diab , B. Al Hussaini , B. Setiabudiawan , U. Kudla , R. J. J. van Neerven , L. Muhardi , J. O. Warner , Front. Immunol. 2021, 12, 1. 10.3389/fimmu.2021.608372 PMC8222906 34177882

[3] A. Fiocchi , A. Bognanni , J. Brożek , M. Ebisawa , H. Schünemann , I. J. Ansotegui , S. Arasi , A. H. Assa'ad , S. L. Bahna , R. B. Canani , M. Bozzola , D. Chu , L. Dahdah , C. Dupont , R. T. Firmino , E. Galli , R. Kamenwa , G. Lack , H. Li , A. Martelli , A. Nowak‐Węgrzyn , N. G. Papadopoulos , R. Pawankar , M. Said , M. Sánchez‐Borges , R. Shamir , J. M. Spergel , H. Szajewska , L. Terracciano , Y. Vandenplas , et al., World Allergy Organ. J. 2022, 15, 10.1016/j.waojou.2021.100609 PMC8818560 35145603

[4] S. Halken , A. Muraro , D. de Silva , E. Khaleva , E. Angier , S. Arasi , H. Arshad , H. T. Bahnson , K. Beyer , R. Boyle , G. du Toit , M. Ebisawa , P. Eigenmann , K. Grimshaw , A. Hoest , C. Jones , G. Lack , K. Nadeau , L. O'Mahony , H. Szajewska , C. Venter , V. Verhasselt , G. W. K. Wong , G. Roberts , Pediatr. Allergy Immunol. 2021, 32, 843. 33710678 10.1111/pai.13496

[5] N. Z. Maryniak , A. I. Sancho , E. B. Hansen , K. L. Bøgh , Foods 2022, 11, 1.

[6] M. Urashima , M. Urashima , H. Mezawa , M. Okuyama , T. Urashima , D. Hirano , N. Gocho , H. Tachimoto , JAMA Pediatr. 2019, 173, 1137. 31633778 10.1001/jamapediatrics.2019.3544 PMC6806425

[7] A. Muraro , T. Werfel , K. Hoffmann‐Sommergruber , G. Roberts , K. Beyer , C. Bindslev‐Jensen , V. Cardona , A. Dubois , G. Dutoit , P. Eigenmann , M. Fernandez Rivas , S. Halken , L. Hickstein , A. Høst , E. Knol , G. Lack , M. J. Marchisotto , B. Niggemann , B. I. Nwaru , N. G. Papadopoulos , L. K. Poulsen , A. F. Santos , I. Skypala , A. Schoepfer , R. Van Ree , C. Venter , M. Worm , B. Vlieg‐Boerstra , S. Panesar , D. De Silva , et al., Allergy Eur. J. Allergy Clin. Immunol. 2014, 69, 1008. 10.1111/all.12429 24909706

[8] S. L. Prescott , M. L. K. Tang , Med. J. Aust. 2005, 182, 464. 15865590 10.5694/j.1326-5377.2005.tb06787.x

[9] P. A. Joshi , J. Smith , S. Vale , D. E. Campbell , Med. J. Aust. 2019, 210, 89. 30636277 10.5694/mja2.12102

[10] D. M. Fleischer , E. S. Chan , C. Venter , J. M. Spergel , E. M. Abrams , D. Stukus , M. Groetch , M. Shaker , M. Greenhawt , J. Allergy Clin. Immunol. Pract. 2021, 9, 22. 33250376 10.1016/j.jaip.2020.11.002

[11] A. von Berg , B. Filipiak‐Pittroff , U. Krämer , E. Link , J. Heinrich , S. Koletzko , A. Grübl , U. Hoffmann , C. Beckmann , D. Reinhardt , C. P. Bauer , E. Wichmann , D. Berdel , Allergol. Sel. 2017, 1, 28. 10.5414/ALX01462E PMC6039995 30402599

[12] Y. Vandenplas , B. Hauser , C. Van den Borre , L. Sacre , I. Dab , Ann. Allergy 1992, 68, 419. 1586005

[13] R. K. Chandra , Nutr. Res. 1998, 18, 1395.

[14] A. J. Lowe , C. S. Hosking , C. M. Bennett , K. J. Allen , C. Axelrad , J. B. Carlin , M. J. Abramson , S. C. Dharmage , D. J. Hill , J. Allergy Clin. Immunol. 2011, 128, 360.e4. 21696814 10.1016/j.jaci.2010.05.006

[15] D. de Silva , S. Halken , C. Singh , A. Muraro , E. Angier , S. Arasi , H. Arshad , K. Beyer , R. Boyle , G. du Toit , P. Eigenmann , K. Grimshaw , A. Hoest , C. Jones , E. Khaleva , G. Lack , H. Szajewska , C. Venter , V. Verhasselt , G. Roberts , Pediatr. Allergy Immunol. 2020, 31, 813. 32396244 10.1111/pai.13273

[16] D. A. Osborn , J. K. H. Sinn , L. J. Jones , Cochrane Database Syst. Rev. 2018, 1. 10.1002/14651858.CD003664.pub6 PMC6517017 30338526

[17] R. J. Boyle , D. Ierodiakonou , T. Khan , J. Chivinge , Z. Robinson , N. Geoghegan , K. Jarrold , T. Afxentiou , T. Reeves , S. Cunha , M. Trivella , V. Garcia‐Larsen , J. Leonardi‐Bee , BMJ 2016, 352, i974. 26956579 10.1136/bmj.i974 PMC4783517

[18] H. Iwamoto , T. Matsubara , T. Okamoto , T. Matsumoto , M. Yoshikawa , Y. Takeda , Int. Arch. Allergy Immunol. 2019, 179, 221. 31030197 10.1159/000497410

[19] K. B. Graversen , J. M. Larsen , S. S. Pedersen , L. V. Sørensen , H. F. Christoffersen , L. N. Jacobsen , S. Halken , T. R. Licht , M. I. Bahl , K. L. Bøgh , Front. Immunol. 2021, 12, 1. 10.3389/fimmu.2021.705543 PMC8438296 34531857

[20] R. Fritsche , Toxicol. Lett. 2003, 141, 303. 10.1016/s0378-4274(03)00026-2 12676478

[21] L. H. Jensen , J. M. Larsen , C. B. Madsen , R. R. Laursen , L. N. Jacobsen , K. L. Bøgh , Int. Arch. Allergy Immunol. 2019, 178, 307. 30759437 10.1159/000495801

[22] A. Chikhi , K. E. Elmecherfi , H. Bernard , N. Cortes‐Perez , O. Kheroua , D. Saidi , K. Adel‐Patient , Pediatr. Allergy Immunol. 2019, 30, 370. 30672606 10.1111/pai.13017

[23] A. Fiocchi , L. Dahda , C. Dupont , C. Campoy , V. Fierro , A. Nieto , World Allergy Organ. J. 2016, 9, 1. 27895813 10.1186/s40413-016-0125-0 PMC5109783

[24] R. Berni Canani , R. Nocerino , G. Terrin , A. Coruzzo , L. Cosenza , L. Leone , R. Troncone , J. Allergy Clin. Immunol. 2012, 129, 580. 22078573 10.1016/j.jaci.2011.10.004

[25] A. Fox , J. A. Bird , A. Fiocchi , J. Knol , R. Meyer , S. Salminen , G. Sitang , H. Szajewska , N. Papadopoulos , World Allergy Organ. J. 2019, 12, 100034. 31194186 10.1016/j.waojou.2019.100034 PMC6555906

[26] K. B. Graversen , A. S. R. Ballegaard , L. H. Kræmer , S. E. Hornslet , L. V. Sørensen , H. F. Christoffersen , L. N. Jacobsen , E. Untersmayr , J. J. Smit , K. L. Bøgh , Clin. Exp. Allergy 2020, 50, 708. 32077177 10.1111/cea.13587

[27] D. Vita , G. Passalacqua , G. Di Pasquale , L. Caminiti , G. Crisafulli , I. Rulli , G. B. Pajno , Pediatr. Allergy Immunol. 2007, 18, 594. 18001430 10.1111/j.1399-3038.2007.00567.x

[28] P. Polidori , S. Vincenzetti , Foods 2013, 2, 151. 28239105 10.3390/foods2020151 PMC5302262

[29] E. M. Navarrete‐Rodríguez , L. A. Ríos‐Villalobos , C. R. Alcocer‐Arreguín , B. E. Del‐Rio‐Navarro , J. M. Del Rio‐Chivardi , O. J. Saucedo‐Ramírez , J. J. L. Sienra‐Monge , R. V. Frias , Allergol. Imunopathologia 2017, 46, 149. 10.1016/j.aller.2017.06.005 29223706

[30] E. Verduci , S. D'Elios , L. Cerrato , P. Comberiati , M. Calvani , S. Palazzo , A. Martelli , M. Landi , T. Trikamjee , D. G. Peroni , Nutrients 2019, 11, 1739. 31357608 10.3390/nu11081739 PMC6723250

[31] G. Docena , P. Rozenfeld , R. Fernández , C. A. Fossati , Allergy Eur. J. Allergy Clin. Immunol. 2002, 57, 83. 10.1034/j.1398-9995.2002.1o3219.x 11929409

[32] K. M. Järvinen , P. Chatchatee , Curr. Opin. Allergy Clin. Immunol. 2009, 9, 251. 19412090 10.1097/ACI.0b013e32832b3f33

[33] M. S. Ehlayel , K. A. Hazeima , F. Al‐Mesaifri , A. Bener , Allergy Asthma Proc. 2011, 32, 255. 21703103 10.2500/aap.2011.32.3429

[34] N. Z. Maryniak , E. B. Hansen , A.‐S. R. Ballegaard , A. I. Sancho , K. L. Bøgh , Nutrients 2018, 10, 1903.30518040

[35] A. Mati , C. Senoussi‐Ghezali , S. Si Ahmed Zennia , D. Almi‐Sebbane , H. El‐Hatmi , J. M. Girardet , Int. Dairy J. 2017, 73, 25.

[36] M. Ehlayel , A. Bener , K. Abu Hazeima , F. Al‐Mesaifri , ISRN Allergy 2011, 2011, 1. 10.5402/2011/391641 PMC3658853 23724227

[37] P. Mudgil , W. N. Baba , M. Alneyadi , A. Ali Redha , S. Maqsood , Lwt 2021, 154, 112813.

[38] A. Omar , N. Harbourne , M. J. Oruna‐Concha , Int. Dairy J. 2016, 58, 31.

[39] A. J. Aburiziza , Curr. Pediatr. Res. 2020, 24, 175.

[40] P. Restani , A. Gaiaschi , A. Plebani , B. Beretta , G. Cagavni , A. Fiocchi , C. Poiesi , T. Velona , A. G. Ugazio , C. L. Galli , Clin. Exp. Allergy 1999, 29, 997. 10383602 10.1046/j.1365-2222.1999.00563.x

[41] P. Restani , B. Beretta , A. Fiocchi , C. Ballabio , C. L. Galli , Ann. Allergy, Asthma Immunol. 2002, 89, 11. 12487198 10.1016/s1081-1206(10)62116-3

[42] E. I. El‐Agamy , M. Nawar , S. M. Shamsia , S. Awad , G. F. W. Haenlein , Small Rumin. Res. 2009, 82, 1.

[43] K. B. Graversen , M. I. Bahl , J. M. Larsen , A. S. R. Ballegaard , T. R. Licht , K. L. Bøgh , Front. Microbiol. 2020, 11, 1. 32292395 10.3389/fmicb.2020.00496 PMC7135894

[44] A. V. Locke , J. M. Larsen , K. B. Graversen , T. R. Licht , M. I. Bahl , K. L. Bøgh , Scand. J. Immunol. 2022, 95, 1. 10.1111/sji.13148 PMC9285443 35152475

[45] K. L. Bøgh , V. Barkholt , C. B. Madsen , Int. Arch. Allergy Immunol. 2013, 161, 21. 10.1159/000343042 23257607

[46] C. L. Thang , X. Zhao , FRIN 2015, 71, 126.

[47] S. T. Hospital , K. Children , 2018, 8555, 259.

[48] B. C. A. M. van Esch , B. Schouten , S. de Kivit , G. A. Hofman , L. M. J. Knippels , L. E. M. Willemsen , J. Garssen , Pediatr. Allergy Immunol. 2011, 22, 820. 21933283 10.1111/j.1399-3038.2011.01205.x

[49] G. Ciprandi , Eur. Ann. Allergy Clin. Immunol. 2015, 47, 62. 25781197

[50] “Circassia Announces Top‐Line Results from Cat Allergy Phase III Study,” https://www.circassia.com/media/press‐releases/circassia‐announces‐top‐line‐results‐from‐cat‐allergy‐phase‐iii‐study/, n.d.

[51] C. L. Thang , X. Zhao , Food Res. Int. 2015, 71, 126.

[52] P. Rupa , Y. Mine , Allergy Eur. J. Allergy Clin. Immunol. 2012, 67, 74. 10.1111/j.1398-9995.2011.02724.x 21950267

[53] M. Yang , C. Yang , Y. Mine , Clin. Exp. Allergy 2010, 40, 668. 20082619 10.1111/j.1365-2222.2009.03442.x

[54] P. Rodríguez del Río , S. Sánchez‐García , C. Escudero , C. Pastor‐Vargas , J. J. Sánchez Hernández , I. Pérez‐Rangel , M. D. Ibáñez , Pediatr. Allergy Immunol. 2012, 23, 128. 22432882 10.1111/j.1399-3038.2012.01284.x

[55] D. Infante Pina , R. Tormo Carnice , M. Conde Zandueta , An. Pediatr. 2003, 59, 138. 10.1016/s1695-4033(03)78737-2 12882742

[56] L. Businco , P. G. Giampietro , P. Lucenti , F. Lucaroni , C. Pini , G. Di Felice , P. Lacovacci , C. Curadi , M. Orlandi , J. Allergy Clin. Immunol. 2000, 105, 1031. 10808187 10.1067/mai.2000.106377

[57] M. S. Motosue , T. Dominquez , A. Sciancalepore , D. Pineda , A. Mehrotra , L. Hoyte , K. Nadeau , J. Allergy Clin. Immunol. 2013, 131, 335.

[58] J. van der Valk , B. Nagl , R. G. van Wljk , B. Bohle , N. de Jong 2020, 1, 1. 10.3390/nu12020519 PMC7071292 32085633

[59] K. S. Hansen , M. S. Khinchi , P. S. Skov , C. Bindslev‐Jensen , L. K. Poulsen , H. J. Malling , Mol. Nutr. Food Res. 2004, 48, 441. 15508179 10.1002/mnfr.200400037

[60] M. Mauro , M. Russello , C. Incorvaia , G. Gazzola , F. Frati , P. Moingeon , G. Passalacqua , Int. Arch. Allergy Immunol. 2011, 156, 416. 21832831 10.1159/000323909

[61] J. Haka , M. H. Niemi , K. Iljin , V. S. Reddy , K. Takkinen , M. Laukkanen , BMC Biotechnol. 2015, 1. 26013405 10.1186/s12896-015-0157-5 PMC4446070

[62] A. Elizur , M. Y. Appel , L. Nachshon , M. B. Levy , N. E.‐ Rigbi , Y. Koren , M. Holmqvist , H. Porsch , J. Lidholm , M. R. Goldberg , Eur. J. Allergy Clin. Immunol. 2022, 77, 1. 10.1111/all.15212 35000223

[63] L. N. Willison , P. Tawde , J. M. Robotham , R. M. Penney IV , S. S. Teuber , S. K. Sathe , K. H. Roux , Clin. Exp. Allergy 2008, 1, 1229. 10.1111/j.1365-2222.2008.02998.x 18479490

[64] A. Pomés , J. M. Davies , G. Gadermaier , C. Hilger , T. Holzhauser , J. Lidholm , A. L. Lopata , G. A. Mueller , A. Nandy , C. Radauer , S. K. Chan , U. Jappe , J. Kleine‐Tebbe , W. R. Thomas , M. D. Chapman , M. van Hage , R. van Ree , S. Vieths , M. Raulf , R. E. Goodman , Mol. Immunol. 2018, 100, 3. 29625844 10.1016/j.molimm.2018.03.003 PMC6019191

[65] I. Sélo , G. Clément , H. Bernard , J. M. Chatel , C. Créminon , G. Peltre , J. M. Wal , Clin. Exp. Allergy 1999, 29, 1055. 10457108 10.1046/j.1365-2222.1999.00612.x

[66] R. N. Pena , J. M. Folch , A. Sánchez , C. B. A. Whitelaw , Biochem. Biophys. Res. Commun. 1998, 252, 649. 9837761 10.1006/bbrc.1998.9718

[67] S. Behrouz , S. Saadat , A. Memarzia , H. Sarir , G. Folkerts , M. H. Boskabady , Front. Immunol. 2022, 13, 1. 10.3389/fimmu.2022.855342 PMC9039309 35493477

[68] P. Sengupta , Int. J. Prev. Med. 2013, 4, 624. 23930179 PMC3733029

