# Supplementary material for: Camel Milk Cannot Prevent the Development of Cow's Milk Allergy—A Study in Brown Norway Rats
Source: Mol Nutr Food Res. 2022 Dec 5;67(2):2200359. doi: 10.1002/mnfr.202200359 (PMC10078016; doi:10.1002/mnfr.202200359)
Supplement: Supplementary file 1 — Supporting Information [file MNFR-67-0-s001.pdf]

Supplementary Figures

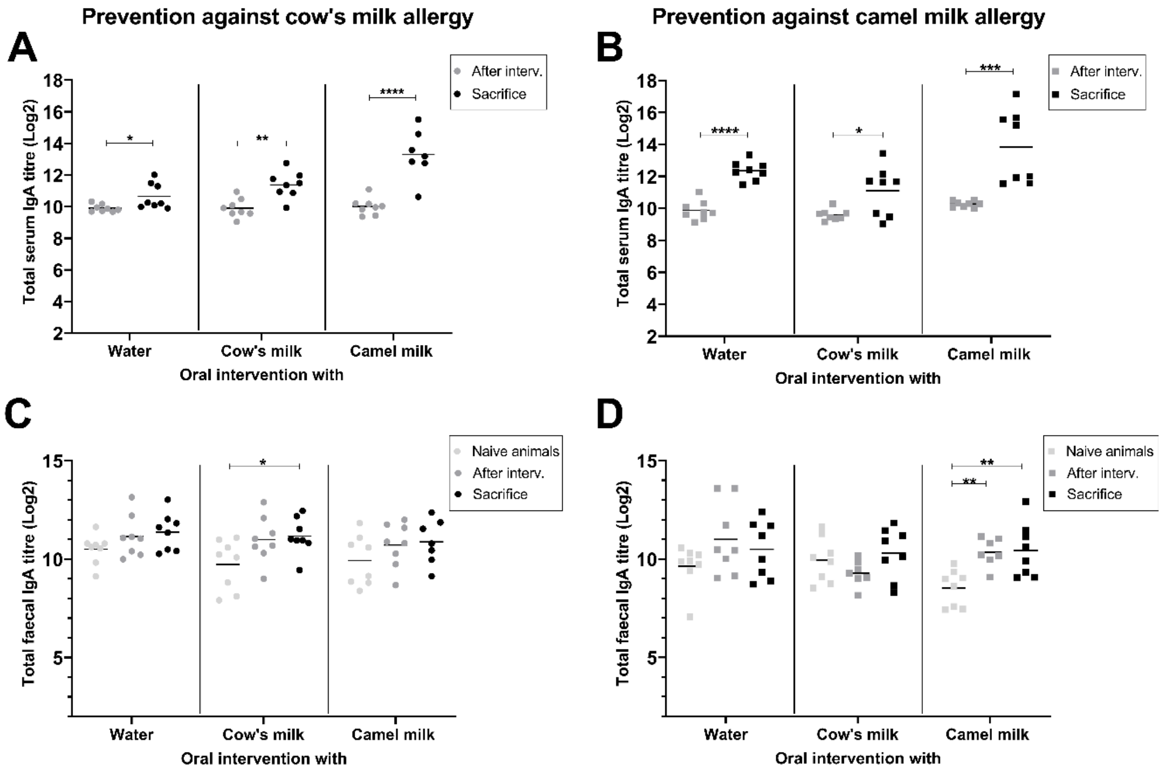

Figure S1. (A) Total IgA in serum from rats intervened with either water, cow's milk or camel milk and tested for prevention against cow's milk allergy. **(B)** Total IgA in serum from rats intervened with either water, cow's milk or camel milk and tested for prevention against camel milk allergy. **(C)** Total IgA in faeces from rats intervened with either water, cow's milk or camel milk and tested for prevention against cow's milk allergy. **(D)** Total IgA in faeces from rats intervened with either water, cow's milk or camel milk and tested for prevention against camel milk allergy**.** Each color represents different time points of the experiment: (●),(■) beginning of experiment (naïve rats), (●),(■) after intervention phase (after interv.) and (●),(■) the day of sacrifice. Either a parametric t-test **(A,B)** or an one-way ANOVA followed by Bonferroni post-test **(C,D)** were applied. Statistically significant differences are shown as *P≤ 0.05, **P≤ 0.01, ***P≤ 0.001 and ****P≤ 0.0001.
